# Efficacy of Activity Trackers in Patients With Heart Failure With Preserved Ejection Fraction

**DOI:** 10.7759/cureus.65117

**Published:** 2024-07-22

**Authors:** Benjamin Kogelschatz, Brittany A Penn, Ashlynn J Leavitt, Elizabeth Dranow, Christy L Ma, John J Ryan

**Affiliations:** 1 Internal Medicine, University of Utah Health, Salt Lake City, USA; 2 Cardiology, University of Utah Health, Salt Lake City, USA

**Keywords:** prospective study, lifestyle intervention, exercise, activity tracker, heart failure with preserved ejection fraction (hfpef)

## Abstract

Background: Heart failure with preserved ejection fraction (HFpEF) is a common, complex syndrome associated with elevated morbidity and mortality. Patients with HFpEF have a high prevalence of comorbidities, including hypertension, diabetes mellitus, and obesity, which are closely related to the underlying mechanisms of the disease. Lifestyle modification with weight loss and physical activity can improve risk factors and functional outcomes in HFpEF. We sought to observe daily physical activity and determine whether utilizing an activity tracker can enhance functional status in HFpEF patients.

Methods: We performed a prospective analysis of 57 patients with HFpEF from 2021 to 2023 at a single academic medical center who utilized a Fitbit to record one year of daily step activity. The patients were evaluated in the ambulatory setting for an initial visit and subsequently at intervals of 3, 6, and 12 months to gather vitals, labs, physical exam, and functional measurements, including the Six-Minute Walk Test (6MWT) and Kansas City Cardiomyopathy Questionnaire-12 (KCCQ-12). Associations between variables were assessed using Pearson’s r correlation using Stata 18.0.

Results: Of the 49 patients who completed the study, the mean age was 68.1 ± 10.2 years, with 67% of patients identifying as female. The average BMI was 36.4 ± 8.6 kg/m^2^. Across each time interval, the median numbers of steps per day were 4,113 (2,517-6,520) (1-3 months), 4,583 (2,532-6,326) (4-6 months), and 3,957 (2,942-5,982) (7-12 months). There was no statistically significant variation in daily step count (p=0.06). We observed a statistically significant increase of 66 (6-200) feet in the 6MWT (p= 0.002) from baseline (1,175 (910-1,400)) to 12 months (1,321 (1,000-1,550)). The daily step count was highly correlated with the 6MWT across all time points (1-3 months: r= .70, p< .001; 4-6 months: r= .61, p< .001; 7-12 months: r= .69, p< .001). The total KCCQ-12 scores increased by 6.8 (-4.2-19.8) points (p=0.005) from baseline (60.1 (41.7-73.4)) to 12 months (69.8 (50-84.4)). Among the sub-categories of the questionnaire, we observed a positive correlation between physical limitation scores and daily step count (1-3 months: r= .47, p=.001; 4-6 months: r= .63, p< .001; 7-12 months: r= .56, p= .001). Of interest, one patient who was taking over 15,000 daily steps scored their physical limitation 10-20 points lower than those taking less than half the steps and had one of the lowest quality of life scores in the cohort, reflecting the subjective nature of heart failure (HF) symptoms.

Conclusion: Fitbit technology offers a convenient means to monitor real-time physical activity in patients with HFpEF. Utilizing a Fitbit to record daily step activity enhances health-related quality of life in this population. In contrast to the improved average total KCCQ-12 score, we did not observe a clinically significant increase in the 6MWT over the course of the year. Our findings establish the utility of daily step count as a valuable surrogate for six-minute walk distance.

## Introduction

Heart failure with preserved ejection fraction (HFpEF) remains a formidable challenge in modern healthcare. It encompasses over half of all patients with heart failure (HF), and its incidence is growing when compared to HF with reduced ejection fraction [1]. Notably, between 2008 and 2018, the number of HFpEF hospitalizations more than doubled [2]. Despite significant therapeutic advancements, the disease continues to carry a substantial burden of morbidity and mortality [3]. The pathophysiology of HFpEF is complex and likely arises from the interplay of multiple mechanisms rather than a singular inciting factor. Patients with HFpEF have a high prevalence of comorbidities including hypertension, diabetes mellitus, and obesity, which appear to be closely related to the underlying mechanisms of the disease [1,4]. In addition to the pharmacologic therapies that have been proven beneficial in the treatment of HFpEF, management of comorbidities is imperative for optimal care of these patients. 

Lifestyle modification with physical activity and weight loss can improve risk factors and functional outcomes in HFpEF. The 2022 American College of Cardiology/American Heart Association (ACC/AHA) Heart Failure guidelines recommend physical activity because of replicated data demonstrating improved quality of life, functional status, and exercise performance [5]. A sedentary lifestyle contributes to most of the risk factors associated with HFpEF. Despite the benefits of physical activity, challenges pertaining to accessibility, adherence, and motivation persist as substantial barriers to improving overall health. Wearable health technologies provide an opportunity to enhance healthy lifestyle behaviors through habit formation and goal adherence. Data in the general population has suggested that activity trackers can lead to increased daily step count and a decrease in body weight [6,7].

In patients with HF, research has demonstrated that tailored, supervised, and progressive exercise programs result in improved physical function and prognostic benefit [8,9]. However, these programs have strikingly low rates of enrollment and participation due to barriers, including availability, travel, willingness to participate, and navigation of the medical system [10]. Walking is recognized as an accessible, sustainable form of exercise which mitigates many of the obstacles patients face with a structured exercise program. Limited data exists regarding the impact of daily walking on functional status in HF patients. We sought to observe daily physical activity and determine whether utilizing an activity tracker can enhance functional outcomes in HFpEF patients.

## Materials and methods

Study population

Investigators enrolled adults diagnosed with HFpEF who were patients at the cardiology clinic at the University of Utah Health between July 2021 and June 2023. IRB approval was obtained from the University of Utah Health. The study recruited patients above the age of 18 who had a confirmed diagnosis of HFpEF and were on standard medical therapy for at least one month prior to enrollment. Patients were diagnosed with HFpEF if they demonstrated dyspnea on exertion or elevated brain-natriuretic peptide (BNP, NT-proBNP) levels in addition to a left ventricular ejection fraction (LVEF) > 45% and diastolic dysfunction on echocardiography. The patients were required to own or have access to a smartphone in order to download the Fitbit mobile application. Patients were ineligible for the study if they had skin damage or significant arthritis preventing them from wearing the device. Other exclusion criteria included patients with the diagnosis of HF with reduced ejection fraction (LVEF <45%) as well as those who were not able to perform daily physical activity or complete the Six-Minute Walk Test (6MWT).

Data collection

The patients were evaluated at the University of Utah Health Cardiovascular Center for clinical visits at baseline, 3 months, 6 months, and 12 months during the study to collect various data. Demographic and clinical data were collected through interviews and questionnaires. Demographic data included age, gender, race, and ethnicity. Clinical data encompassed date of HF diagnosis, LVEF on echocardiography, medications (specifically angiotensin-converting enzyme inhibitor/angiotensin II receptor blockers, beta-blockers, diuretics, statins, aspirin), New York Heart Association (NYHA) class, physical exam findings, laboratory values (BNP, NT-proBNP), 6MWT, and the Kansas City Cardiomyopathy Questionnaire-12 (KCCQ-12). The clinical data was obtained at each of the four patient encounters throughout the year.

At the baseline visit, patients were provided with a wrist-worn Fitbit activity tracker to monitor their daily step count. Patients were instructed to wear the device for the entire day. They were given no specific instructions regarding how to change their activity level when wearing the activity tracker. We utilized the software Fitabase to collect data from each Fitbit device. Patients were provided education on how to sync their device to the database from our study coordinators. The patients were able to contact the study coordinators at any point if they experienced issues with utilizing their Fitbit. If the patient had not synced their data in roughly one week, the study coordinators would contact the patient to encourage syncing or assist in troubleshooting any technical issues. Average steps were calculated by month using only the days where the Fitbit was worn, as indicated by other data collected by the device.

Outcomes

The two primary outcomes were the difference between the baseline and 12-month values of both the KCCQ-12 scores and distance walked during the 6MWT. Secondary outcomes included change in daily step count, correlation between daily step count and 6MWT, sub-categories of the KCCQ-12 (quality of life, physical limitation, social limitation, and symptom frequency), biomarkers (NT-proBNP, BNP), and BMI.

Statistical analysis

Baseline characteristics were reported using basic summary statistics, including mean and standard deviation or median and interquartile range for continuous variables, as appropriate, and percentages for categorical variables. The Pearson correlation coefficient (r) was used to assess the strength and direction of the linear relationship between daily step count, 6MWT, and KCCQ-12 questionnaire scores at each time interval (1-3 months, 4-6 months, 7-12 months). A p-value was calculated to establish the statistical significance of the correlations. The Wilcoxon matched-pairs signed-rank test was used to compare KCCQ-12 questionnaire, average steps, and 6MWT results between baseline and the other time intervals. The p-value was determined with a two-tailed significance level set at 5%. The primary outcome is reported as median and interquartile range. All analyses were conducted using STATA 18.0 (StataCorp LLC, College Station, TX, USA).

## Results

49 patients completed a full year of data collection with the Fitbit device. Seven patients withdrew from the study, and one patient died during the trial period. In the cohort, the mean age was 68.1 ± 10.2 years with 33 (67.3%) of the patients identifying as female. The average BMI was 36.4 ± 8.6 kg/m^2^. 44 (89.9%) patients were Caucasian. The average ejection fraction was 64.2 ± 5.2%. 38 (77.5%) of the patients were classified as NYHA Class II, and the remaining 11 (22.4%) were NYHA Class III. The median number of months since HFpEF diagnosis was 31.4 (11.8-69.8) (Table 1).

**Table 1:** Baseline characteristics Baseline characteristics are reported from the patients who completed the full study. HF: Heart failure; IQR: Interquartile range; NYHA: New York Heart Association; ACEi: Angiotensin-converting enzyme inhibitor; ARB: Angiotensin II receptor blocker; BNP: Brain natriuretic peptide; NT-proBNP: N-terminal-pro brain natriuretic peptide; 6MWT: Six-Minute Walk Test; KCCQ-12= Kansas City Cardiomyopathy Questionnaire-12

Characteristics		All patients (n= 49)
Age (years)		68.1 ± 10.2
Sex		
Female		33 (67.3%)
Male		16 (32.7%)
Race		
Caucasian		44 (89.8%)
African American		1 (2.0%)
Other		4 (8.2%)
BMI (kg/m^2^), mean ± SD		36.4 ± 8.6
HF duration (months), median (IQR)		31.4 (11.8-69.8)
Ejection fraction (%), mean ± SD		64.2 ± 5.6
NYHA Class		
II		38 (77.5%)
III		9 (18.4%)
Medication		
Beta-blocker		17 (34.7%)
ACEi		9 (18.4%)
ARB		23 (46.9%)
Diuretic		43 (87.8%)
Statin		30 (61.2%)
Aspirin		15 (30.6%)
Blood pressure		
Systolic		126.6 ± 14.6
Diastolic		76.5 ± 8.2
BNP (pg/mL), median (IQR)		52 (14-96)
NT-proBNP (pg/mL), median (IQR)		144 (77-248)
6MWT (feet), median (IQR)		1175 (910-1400)
KCCQ-12 total score, median (IQR)		63.5 (42.7 – 73.7)

**Figure 1:**
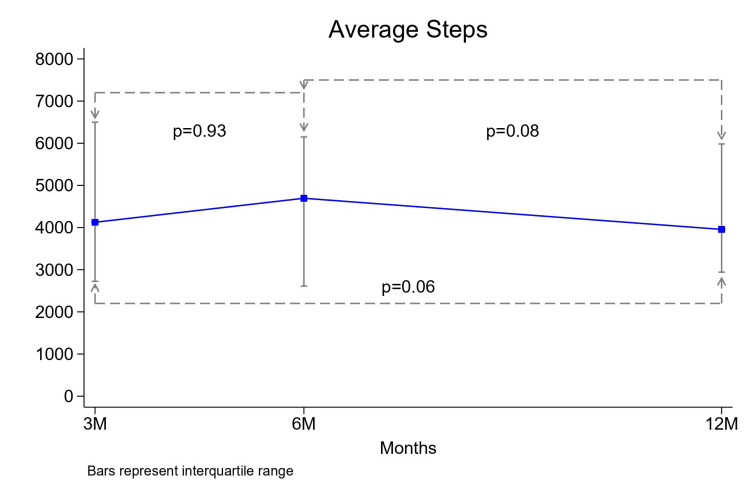
Average daily steps The median daily steps measured at 3 months (3M), 6 months (6M), and 12 months (12M) are displayed as a line graph. Data points signify median values at each time point, with error bars representing interquartile range. p-values indicate the statistical significance of differences in daily step count between time points.

Across each time interval, the median steps per day were 4,114 (2,517-6,520) (1-3 months), 4,583 (2,532-6,326) (4-6 months), and 3,957 (2,943-5,983) (7-12 months). There was no statistically significant variation in step count throughout the year (p= 0.06) (Figure 1).

**Figure 2:**
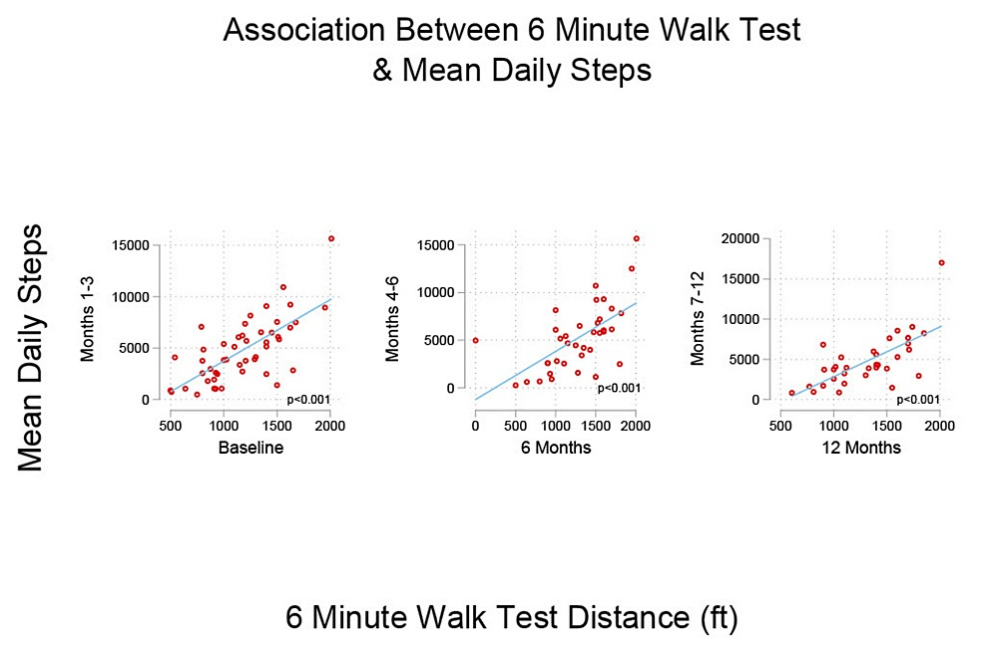
Association between 6MWT and mean daily steps Three scatter plots demonstrate the linear relationship between the 6MWTdistance (in feet) and mean daily steps over three time intervals (1-3 months, 4-6 months, and 7-12 months). 6MWT: Six-Minute Walk Test

The daily step count was highly correlated with the 6MWT across all time points (1-3 months: r= .70, p< .001; 4-6 months: r= .61, p< .001; 7-12 months: r= .69, p< .001) (Figure 2).

**Figure 3:**
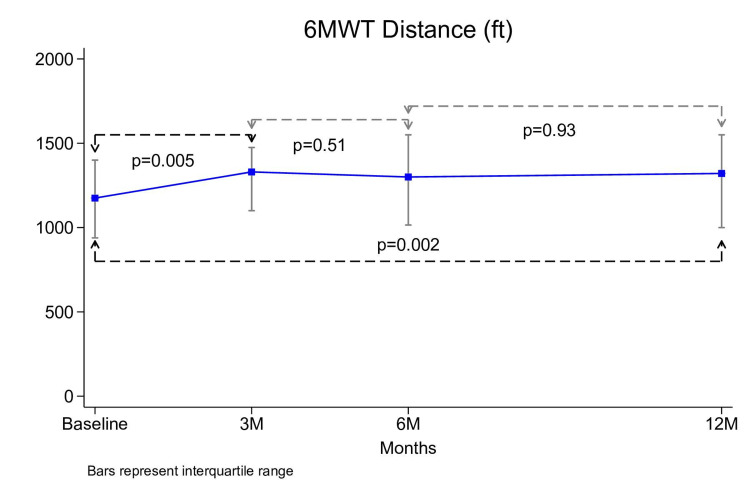
Trend in 6MWT distance The median 6MWT distance (in feet) measured at baseline, 3 months (3M), 6 months (6M), and 12 months (12M) is shown as a line graph. Data points signify median values at each time point, with error bars representing the interquartile range. p-values indicate the statistical significance of differences in 6MWT distances between the time points. 6MWT: Six-Minute Walk Test

The mean 6MWT was 1175 (910-1,400) feet at baseline, 1,375 (1,050-1,539) feet at 3 months, 1,313 (1,000-1,550) feet at 6 months, and 1,321 (1,000-1,550) feet at 12 months. From baseline to 12 months, the change in 6MWT was a statistically significant increase of 66.2 (6-200) feet (p= .002) (Figure 3).

**Figure 4:**
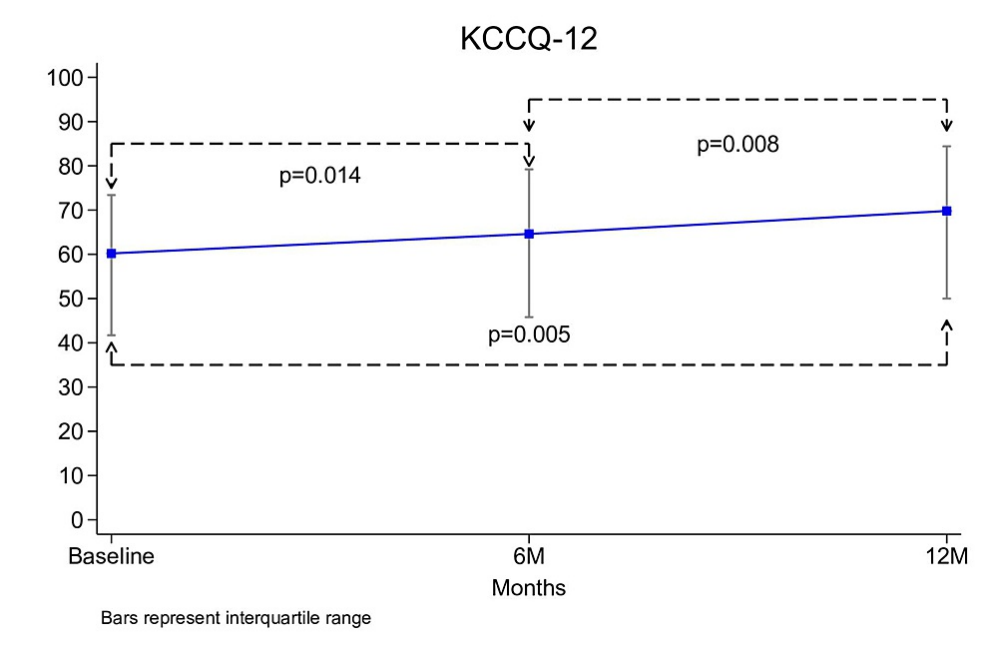
Trend in KCCQ-12 The line graph demonstrates the median KCCQ-12 scores at baseline, 6 months (6M), and 12 months (12M). Error bars represent the interquartile range. p-values indicate the statistical significance of differences in KCCQ-12 scores between time points. KCCQ-12: Kansas City Cardiomyopathy Questionnaire-12

The total KCCQ-12 questionnaire scores increased by 9.6 points from baseline to 12 months, from 60.2 (41.7-73.4) to 69.8 (50.0-84.4) (p=.005) (Figure 4).

Among the sub-categories of the questionnaire, we observed a positive correlation between physical limitation scores and daily step count (1-3 months: r= .47, p=.001; 4-6 months: r= .63, p< .001; 7-12 months: r= .56, p= .001). The symptom frequency, social limitation, and quality of life sub-categories had no statistically significant correlation with daily step count at any time point. BMI had no significant change throughout the course of the year. The median NT-proBNP decreased from 144 (77-348) at the baseline visit to 138 (84-353) at 12 months, although this did not meet statistical significance (p=0.41). Of interest, one patient who was taking over 15,000 daily steps scored their physical limitation 10-20 points lower than those taking less than half the steps and had one of the lowest quality of life scores in the cohort, reflecting the subjective nature of HF symptoms.

## Discussion

Our single-center study investigated the utility of an activity tracker to monitor daily step count and observe its correlation with functional status in patients with HFpEF. The change in 6MWT from baseline to 12 months did reach statistical significance. However, evidence indicates that an increase of over 50 meters may signify clinically meaningful improvement in 6MWT [11]. The total KCCQ-12 scores demonstrated a significant increase from baseline to 12 months indicating a perceived improvement in health-related quality of life. Other HFpEF trials have demonstrated more modest improvement in KCCQ scores following an intervention. For instance, the PARAGON-HF trial studying sacubitril-valsartan showed that only 33% of patients experienced an increase in their KCCQ score by more than five points [12]. Regarding secondary outcomes, we observed a strong positive correlation between the 6MWT and daily step count at each time interval. The daily step count also demonstrated a positive correlation with the physical limitation sub-category of the KCCQ-12. The symptom frequency, quality of life, and social limitation sub-categories of the questionnaire had no correlation to step count. Other measurements including BMI, NT-proBNP, and BNP showed no statistically significant change from baseline to 12 months.

The mean daily step count throughout the year demonstrated no statistically significant variation. However, we observed a nearly significant decrease in median daily step count over the latter half of the year (months 7-12). This could be partly explained by the hypothesis that physical activity levels decrease during the winter months. Based on when patients were enrolled in the study, 32 of the 49 patients had at least 3 months of winter in their final 6 months of the data collection period. These 32 patients demonstrated a decreased median daily step count for months 7-12 compared to the 17 patients who had no winter months during the latter half of the data collection period, although this did not meet significance.

Our findings suggest the utility of daily step count as a valuable surrogate for 6MWT, which has the potential to offer numerous advantages for patients and providers. The 6MWT is routinely conducted in a clinical setting and provides a snapshot of functional capacity at a specific point in time. Activity trackers, on the other hand, enable continuous monitoring of physical activity levels over time which allows for a comprehensive assessment of habitual activity patterns. Longitudinal tracking can help facilitate the detection of trends, fluctuations, and responses to interventions. Additionally, data from an activity tracker can easily be transmitted to facilitate remote monitoring which improves patient and provider convenience. Incorporating daily step count data could empower patients to actively engage in their health management, set personalized goals, and make lifestyle modifications.

Despite these theorized benefits, whether home-based walking has any effect on functional outcomes in patients with HF has not been fully established. In a randomized controlled trial of a 6-month walking intervention in patients with stable HF with reduced ejection fraction, the investigators found no significant increase in 6MWT despite a roughly 25% increase in daily steps in the intervention group. The investigators hypothesized that the pattern, duration, and intensity of the daily physical activity may have been insufficient to influence the 6MWT [13]. REACH-HF is another randomized controlled trial which facilitated a self-care and home-based progressive walking program in patients with HF. The researchers observed no difference in physical activity levels or exercise capacity via the incremental shuttle walk test despite an increase in health-related quality of life in the intervention group [14]. These studies align with our results and indicate that implementing a structured exercise program may be required to enhance functional status in HF patients. The BE ACTIVE trial used activity trackers to monitor patients who were encouraged to become more active through the use of incentives, both behavioral and financial. Each approach provided durable increases in physical activity, even upon withdrawal of the interventions. The authors inferred that the degree of sustained physical activity observed in the trial would translate to a 10% lower risk of cardiovascular mortality [7].

Similar to the REACH-HF trial, it is noteworthy that despite no significant improvement in step count or 6MWT, the KCCQ-12 total score increased from baseline to 12 months in the cohort. One plausible explanation is the patients in this study had more accessibility to the medical system than they ordinarily would. In addition to visiting with a physician at the baseline visit and subsequent 3-, 6-, and 12-month visits, patients were able to reach our study coordinators to help resolve technical issues. As a result, this may have improved emotional support and feelings of isolation regarding their health. In addition, it is possible that there is a perceived health benefit solely from wearing the Fitbit device despite no actual alteration in physical activity. One study investigating the behavioral effects of wearing an activity tracker found that people felt more motivated to improve their overall health, and over 80% of users believed they increased their amount of daily physical activity while wearing a device [15].

Utilizing an activity tracker to monitor daily physical activity in HFpEF patients presents several drawbacks. With longitudinal use, these devices will inherently have technical issues which was demonstrated in the majority of patients in the cohort. Some individuals may also struggle with the technology and require a certain level of health literacy to comprehend the benefits and function of activity trackers. Physical limitations can hinder the ability to easily wear the device and interact with its small buttons or touchscreens. Furthermore, cost remains a barrier to access for many patients. Several patients in our study who had minimal technological issues struggled with compliance due to various reasons including disinterest, forgetfulness, or unrelated health issues which prevented daily walking.

Technical complications were frequent in the cohort in addition to instances of user error. Over the course of the study period, the study coordinators assisted with syncing to all participants on at least one occasion. Notably, five individuals were sent a replacement charger either because they were unable to effectively clean the device to allow proper connection with the charger, or because the charger was misplaced. Furthermore, technical problems prompted the complete replacement of three Fitbit devices. Additionally, two participants opted to withdraw from the study due difficulties operating the device.

There are several limitations of our study. This was a single-center investigation with a limited cohort size, which diminished the generalizability to broader populations and statistical power to detect meaningful differences in outcomes. A portion of the collected data from the Fitbit device was not utilized as there were several patients who had numerous recorded days of zero steps due to poor compliance or technological difficulties. Additionally, there was no control or sham group for comparison. Our study was susceptible to patient expectation bias given the lack of blinding.

## Conclusions

Our study is unique in identifying daily step count as a surrogate for the 6MWT in patients with HFpEF. This presents several advantages including convenience, accessibility, and observation of habitual patterns. Daily use of an activity tracker translated to an improved health-related quality of life despite no significant improvement in daily step count or 6MWT. These findings corroborate previous data demonstrating the challenge heart failure patients face in improving functional status without enrollment in a structured, rigorous exercise program. Our study highlights the need for further investigation into the role that wearable health devices play in improving physical activity and functional status in patients with HFpEF.
